# Mitochondrial Supercomplexes: Physiological Organization and Dysregulation in Age-Related Neurodegenerative Disorders

**DOI:** 10.3389/fendo.2020.00600

**Published:** 2020-09-11

**Authors:** Gisela V. Novack, Pablo Galeano, Eduardo M. Castaño, Laura Morelli

**Affiliations:** Laboratory of Brain Aging and Neurodegeneration-Fundación Instituto Leloir-IIBBA-CONICET, Buenos Aires, Argentina

**Keywords:** supercomplexes organization, respirasome structure, neurodegeneration, Alzheimer's disease, Parkinson's disease, mitochondrial dysfunction, brain bioenergetics

## Abstract

Several studies suggest that the assembly of mitochondrial respiratory complexes into structures known as supercomplexes (SCs) may increase the efficiency of the electron transport chain, reducing the rate of production of reactive oxygen species. Therefore, the study of the (dis)assembly of SCs may be relevant for the understanding of mitochondrial dysfunction reported in brain aging and major neurodegenerative disorders such as Alzheimer's disease (AD) and Parkinson's disease (PD). Here we briefly reviewed the biogenesis and structural properties of SCs, the impact of mtDNA mutations and mitochondrial dynamics on SCs assembly, the role of lipids on stabilization of SCs and the methodological limitations for the study of SCs. More specifically, we summarized what is known about mitochondrial dysfunction and SCs organization and activity in aging, AD and PD. We focused on the critical variables to take into account when postmortem tissues are used to study the (dis)assembly of SCs. Since few works have been performed to study SCs in AD and PD, the impact of SCs dysfunction on the alteration of brain energetics in these diseases remains poorly understood. The convergence of future progress in the study of SCs structure at high resolution and the refinement of animal models of AD and PD, as well as the use of iPSC-based and somatic cell-derived neurons, will be critical in understanding the biological relevance of the structural remodeling of SCs.

## Introduction

Mitochondria are dynamic organelles that reorganize under stress or variations in the availability of nutrients or oxygen. It was proposed that an equilibrated distribution between individual mitochondrial respiratory complexes (MRC) and supercomplexes (SCs) is relevant to achieve the optimal performance of the electron transfer chain. However, the procedures by which the assembly of SCs can be adapted to the requirements of the cells are still poorly understood. In this mini review we address the organization of SCs in cell cultures and brain tissue from human or animal models of aging, AD and PD to evaluate the possible relevance of the (dis)assembly of SCs in the energetic failures characteristics of these neurodegenerative disorders associated with amyloid deposition.

## Biogenesis and Structural Properties of SCs

**Figure 1:**
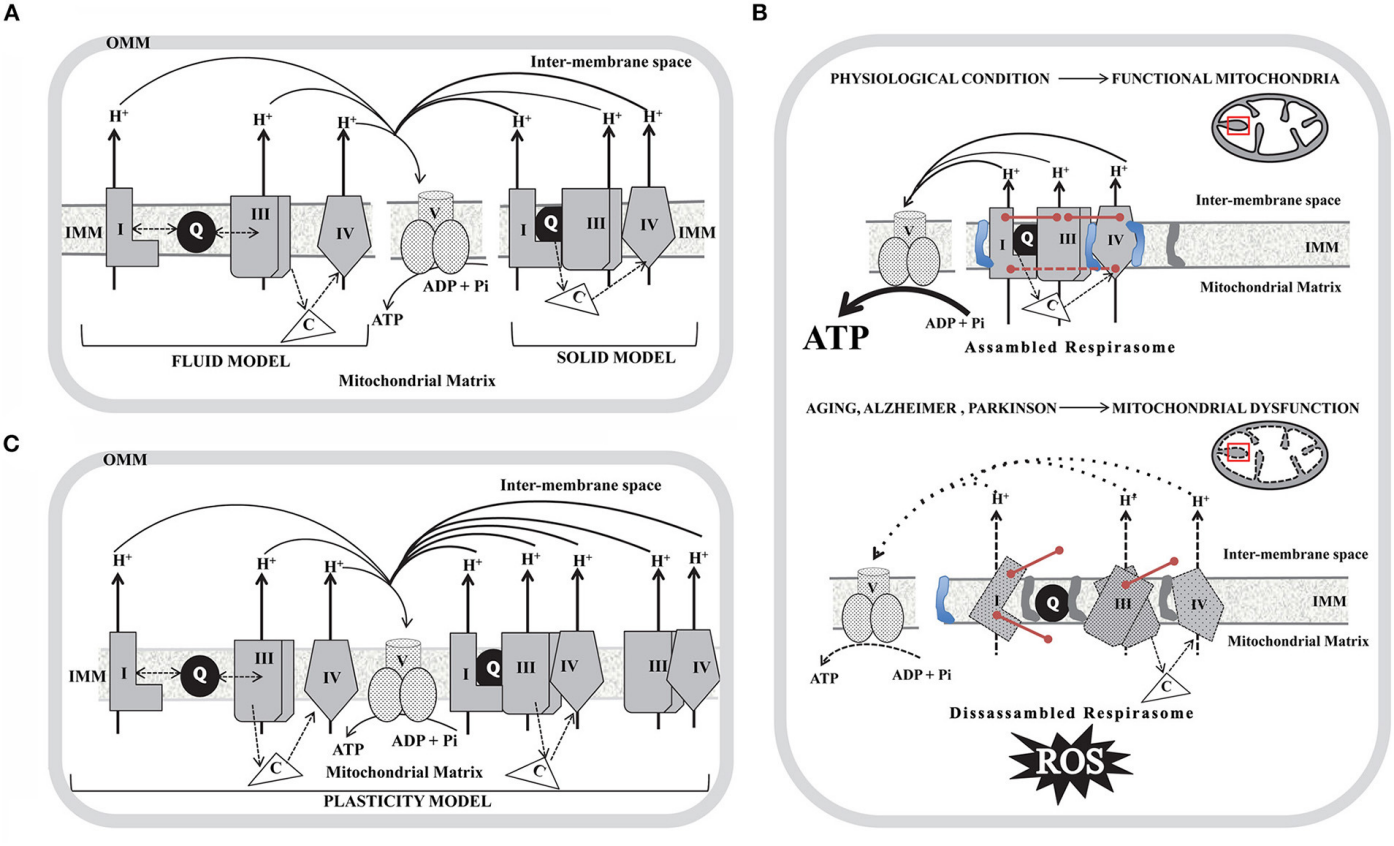
Schematic diagram of the models proposed to explain the organization of the mitochondria respiratory complexes in the inner mitochondrial membrane (IMM) and the impact of the SCs disassembly in aging, Alzheimer and Parkinson disorders. **(A)** Fluid model (left); Solid model (right). **(B)** Plasticity model in which respirasomes exist in dynamic equilibrium with randomly organized, enzymatically active, and isolated complexes. In **(A)** and **(B)** only individual complexes (CI, CIII, and CIV) that conform respirasomes were depicted and do not show the actual stoichiometry; coenzyme Q (Q) is represented as a black-filled circle in the IMM and cytochrome c **(C)** as a non-membrane associated white triangle that transfer electrons from CIII to CIV (dotted arrows). Doble head dotted arrows indicate lateral diffusion of Q between membrane embedded complexes in the Fluid and Plasticity models, while in the Solid model, SCs trap the soluble electron carrier and restrict its diffusion. Solid black arrows show the proton pumping activity of each individual complex and the ATP generation by complex V (CV). OMM, outer mitochondrial membrane. **(C)** In a physiological condition (upper panel) mitochondria show assembled SCs located in intact cristae (depicted as a black line inside the red box in the scheme of mitochondria). SCs integrity is mediated by assembly factors between individual units from each complex (described as pink lines) and by high levels of cardiolipin (represented as a blue structure in the IMM) and by low levels of phosphatidylethanolamine (represented as a gray structure in the IMM). Assembled respirasomes limit ROS generation while improving the efficiency of ATP synthesis. In aging, Alzheimer and Parkinson (lower panel) the dysfunctional mitochondria show dissessembled SCs located in swelled mitochondria cristae (depicted as a dotted black line inside the red box in the scheme of mitochondria). Respirasomes disorganization may be due to impaired assembly factors expression (described as un-connected pink lines) and to low levels of cardiolipin and high levels of phosphatidylethanolamine. Disassembled respirasomes promote ROS generation and decrease ATP levels.

The respiratory chain in mammals consists of four respiratory complexes (I–IV) encoded by nuclear and mitochondrial DNA (mtDNA) inserted and assembled in the inner mitochondrial membrane (IMM) and two intermediary substrates (coenzyme Q and cytochrome c). According to their enzymatic activities complex I (CI) is known as NADH:ubiquinone oxidoreductase, complex II (CII) as succinate:quinone oxidoreductase; complex III (CIII) as ubiquinol-cytochrome c oxidoreductase and complex IV (CIV) as cytochrome *c* oxidase (COX). During the last 30 years, the structural organization of the respiratory complexes was intended to be understood in terms of two utmost paradigms: the “fluid” and the “solid” models. The “fluid” model postulates that all redox components are unconstrained diffusible particles with the electron carriers alternating in the middle of the gigantic complexes I–IV (1, 2). This interpretation suggests that mitochondrial electron transfer is a diffusion-based stochastic collision process and that diffusion has an essential and regulating effect on electron transfer (3). The “fluid” model was accepted until 2001 when it was shown that it was possible to purify, by means of Blue-Native polyacrylamide gel electrophoresis (BN-PAGE), stable associations of respiratory complexes (4) and therefore the “solid” model was proposed. In the “solid” model, respiratory complexes establish interactions to form higher-order supramolecular structures called supercomplexes (SCs) formed by two units of CIII and a variable number of CIV in the presence or not of one module of CI, (I_1_III_2_IV_1−2_; III_2_IV_1−2_), or even into megacomplex where two units of CI, CIII, and CIV get together (I_2_III_2_IV_2_)_._ Based on BN-PAGE, CII was not detected in SCs (5). However, CII could bind to SCs by weak protein-protein interactions that may dissociate due to dilution during mitochondrial isolation. Recent cryo-EM and cross-linked mass spectrometry studies suggested that CII can be involved in respirasomes (6, 7). By contrast, experiments performed in cell cultures demonstrated that silencing a CII subunit (*SDHC*) has no effect on the respirasome assembly, although it can play a regulatory role in respirasome formation (8). In the solid model, SCs I_1_III_2_IV_n_ that act as single functional entities are known as “respirasomes” which are capable of catalyzing whole reaction pathways by the presence of Q and cyt c associated with these devices. An integrative vision of both models was recently proposed, noted as the “plasticity” model (9). This model consolidates transient assemblies and free lateral diffusion of redox units. Both SCs and the free individual components can perform their actions, with SCs being more efficient in energy production with a lower ROS formation rate. However, this paradigm was suggested based on distinctions in the steady-state amounts of respiratory chain complexes analyzed by BN-PAGE technique, and consequently, the proposed “plasticity” between the individual complexes and the respirasomes *in vivo* has not yet been proven. A schematic diagram of the three models is depicted in Figures 1A,B. Additionally, the association of single complexes to form the respirasomes can be explained by two mechanisms. The first proposes that CI gets fully-assembled prior to its binding to SCs, while the second favors the sequential binding of CIII_2_ and CIV to an almost complete CI scaffold (10). Taking into account that “free CI” is underrepresented in mammalian tissues under mild purification conditions, it was proposed that SCs provide a scaffold for the full-assembly and stability of CI (11). 3D maps show extensive and stable interactions between specific CI and CIII_2_ (12–14) and as CI and CIII_2_ subunits contain disulfide bonds it was proposed that redox status may modulate CI-CIII_2_ interactions. A close association between specific CIV and CI subunit (12, 13), and between CIV and CIII_2_ subunits, was also determined (13). Models of SCs from ovine/bovine/porcine mitochondria suggest that “species-specific” protein sequences are critical for the arrangement of SCs subunits. Moreover, it was demonstrated that different cell types from human and mouse strains show similar SCs composition. However, the composition of the minimal SCs (In + IIIn) was variable in different cell types (15). Since some of the units of single complexes are encoded in mtDNA, the impact of mtDNA mutations on SCs assembly was analyzed in human cybrids containing mutations in CIV and CIII subunits. Based on these studies it was proposed that CIII may be the structural core of SCs while CI, because of its instability, cannot exist as an individual entity (16). The role of SCs assembly proteins COX7A2L and respiratory complex factor (Rcf) was also explored (17, 18). It was postulated that COX7A2L may be a regulatory checkpoint for the biogenesis of CIII_2_ and CIII-containing SCs (19) while Rcf1 and Rcf2 are CIV-binding proteins that may also interact with CIII_2_ (20). In contrast, MCJ/DnaJC15 (a co-chaperone localized at the IMM) has been proposed as a negative regulator of SCs formation/stability (21). Even if MCJ is non-essential for mitochondrial function under physiological conditions, MCJ failure impacts on the pathophysiology arising specific metabolic alterations (21).

## Role of Lipids on Stabilization of SCs

Compared to other sub-cellular membranes, the IMM is highly enriched in phosphatidylethanolamine (PE) and cardiolipin (CL), being the latter an evolutionarily conserved dimeric phospholipid that is precisely located in mitochondria. Data of mammalian samples indicate asymmetric phospholipid location in the IMM leaflets, with particular enrichment of phosphatidylinositol (PI) and CL in the matrix-facing leaflet of the IMM (22). *In vitro* and *in vivo* studies highlight the role of PE and CL in the assembly and activity of MRC with the special role of CL in the SCs stabilization and PE in the SCs destabilization (23). In agreement with these remarks, the atomic structure of the respirasomes showed gaps between CI, CIII_2_, and CIV that were immersed by CL to fix properly the respirasomes (24, 25). The functional importance of CL in SCs stabilization is reinforced by studies on cells from patients with a hereditary disease known as Barth syndrome (BTHS) (an X-linked recessive disorder due to the lack of tafazzin, the transacylase that catalyzes the maturation of CL into its polyunsaturated forms). Mitochondria from BTHS lymphoblasts show reduced abundance and instability of SCs which in turn results in a higher rate of CL degradation (26, 27). CL and PE are synthesized in mitochondria from the precursors Cytidine Diphosphate (CDP)- diacylglycerol and CDP-ethanolamine, respectively, both located in the endoplasmic reticulum (ER). The importation of precursors into the mitochondria requires the juxtaposition of ER-mitochondria membranes. This collocation, known as “lipid-Mitochondria-ER Contacts” (“lipid-MERC”), is needed to be properly assembled and functionally operational to guarantee the correct transport between organelles (28). The gap width needed for lipid transfer is anticipated to be as thin as ≤ 10 nm (29) and it has been proposed that disturbances in the interphase length of apposition and the gap width between these organelles may impair lipid exchange (30). Recently, we experimentally addressed this issue in primary neuronal cultures from McGill-R-Thy1-APP transgenic (Tg) rats, a model of the early stage of Alzheimer-like amyloid pathology (31). We found, using *in vivo* FRET and transmission electron microscopy (TEM), significant decrements in the average length of the apposition of “lipid-MERC” (gap width ≤ 10 nm) as compared to control cells, suggesting “relaxed” contacts between organelles. In accordance with FRET and TEM results, untargeted lipidomics of isolated mitochondria from neurons exhibited in Tg a significant deviation. The total number of lipids detected was 106 and 12 were significantly different in Tg as compared to control rats. Among them, a 60% decrement in mitochondrial CL and PE was observed (32). These results allow us to speculate that at early stages of AD pathology, there could be a disassembly of the SCs of neuronal mitochondria due to alteration in CL and PE synthesis, which in turn may promote neuronal bioenergetics dysfunction, a characteristic feature of this neurological disorder (33–36).

## Functional Roles of SCs

The biological significance of respirasomes in oxidative phosphorylation (OXPHOS) has not yet been established. It was proposed that: (1) CIII and CIV association with CI may favor the assembly and stability of CI (37–40). Different studies have shown that the integrity of CIII_2_ and CIV is crucial for the stability of mammalian CI. Even though, CI dysfunction is infrequent in most of the patients with CIII_2_ or CIV enzymatic deficiencies, which is consistent with the possibility that major structural alterations of CIII_2_ and CIV are necessary to induce CI dysfunction (41, 42); (2) The assembly of SCs may reduce the rate of ROS generation (43, 44). CI and CIII_2_ are the major redox centers in which oxygen is reduced to superoxide and, therefore, their assembly into SCs may minimize ROS production. Studies using bovine heart mitochondria have shown that the disassembly enhanced the release of superoxide from CI, and a direct correlation between free CI and ROS generation was found in neurons and astrocytes (45); (3) SCs may optimize the catalytic activity of the individual complexes (46, 47). In this regard, it was shown that the activity of CI in SCs I+III_2_ isolated from bovine heart mitochondria was half of the activity of CI in SCs I+III_2_+IV_1_, indicating that the full respirasome was the most active unit. It has been proposed that the assembly of CI with CIII_2_ favors electron transfer through Q by channeling between the two complexes without following a pool behavior. This proposal has been challenged by studies showing that NADH and succinate oxidation include different Q redox steady states, but communicate and converge on a single non-partitioned Q pool (48, 49); (4) SCs can boost the efficiency of electron transfer through substrate channeling (5, 50, 51). However, the lack of a confining structure between CI and CIII (49) is inconsistent with such a mechanism. A recent study showed, by using inverted sealed vesicles from bovine heart IMM, that the SCs do not sequester or channel the Q pool which is freely exchanged within and outside the SCs sets to react with any enzyme in the membrane (52). Although these experiments were performed in a non-physiological solution lacking the mitochondrial environment and interaction pathways, more physiologically relevant studies carried out in Drosophila (53) and mice (54) also agree with these results; (5) SCs may prevent aggregation of their protein subunits. The IMM is characterized by a high protein:lipid ratio and, therefore, the correct assembly of SCs may be crucial in minimizing the possibility of nucleation and non-functional aggregation of their components (48).

## Methodological Limitations for the Study of SCs

During the last 20 years, various techniques have been implemented for the study of SCs. The first evidence for the existence of SCs was supported by BN-PAGE experiments (5) and more recently, data obtained using cryo-EM provided more crucial information about the structural composition of SCs at near-atomic resolution (12, 55, 56). However, these techniques have several limitations to elucidate the interactions between individual complexes and the mechanisms by which SCs are assembled. Major drawbacks are based on the following issues: (1) the procedure of mitochondria isolation and purification may impact on the SCs integrity (57). It appears that detergent solubilization of SCs may affect their native structure. Variations in the type of detergent used and in the ratio of detergent/mitochondrial protein have a significant effect on the characterization of SCs at the quantitative and qualitative levels; (2) the absence of lipids (especially CL) in the isolation buffer for solubilization of mitochondrial proteins is an additional factor that can promote dissociation of poorly linked complexes; (3) interpretation of the bands resolved by BN-PAGE is not an easy task due to overlapping of signals. Moreover, determination of molecular mass of SCs may be difficult using BN-PAGE due to conformational and surface changes. For the resolution of subunits of protein complexes, the most useful technique is to combine BN-PAGE with a second gel dimension (2D)-BN/sodium dodecyl sulfate (SDS)-PAGE (58). It is of note that SCs in BN-PAGE are highly prone to diffusion during the equilibration step before loading onto the second dimension, generating possible distortions in the densitometry analysis of the spots. In addition to BN-PAGE, the CN-PAGE or “clear native gels” (in the absence of the dye Coomassie Blue) are frequently used to assess ATP synthase in-gel activity. CN-PAGE allows better enzymatic preservation and limits the dissociation of labile proteins in high molecular weight SCs and ATP synthase assemblies (59).

Studies that solubilize SCs without detergents by cross-linking and mass spectrometry (MS) have shown the existence of many *in situ* interactions in proteins throughout electron transfer complexes, ATP synthase, and the mitochondrial contact site and cristae organizing system (60). Recently, it was shown that all respiratory complexes (including CII and CV that were not found by means of cryo-EM) are in close spatial proximity. This provides direct evidence for SCs assembly in the intact mitochondria (7). The heterogeneity in the preparations and particles selected for cryo-EM in different studies may introduce a bias that deserves further clarification (12, 49, 55, 56, 61).

In addition, novel approaches to detect SCs in eukaryotic cells *in vivo* are being developed. In this regard, by using proximity-dependent labeling followed by MS it is possible to identify potentially interacting proteins and their subcellular spatial localization, overcoming the technical difficulties that are associated with transient protein–protein interactions detection (62–64). Moreover, fluorescence lifetime imaging microscopy (FLIM), that employs fluorescent sensor proteins to assess the assembly of SCs and to monitor SCs plasticity in live cells, is currently implemented (65). Recently, Förster Resonance Energy Transfer (FRET) using CIII subunit k fused to Clover as a donor and CoxVIIIa-mRuby2 as an acceptor showed colocalization of CIII and CIV and SCs formation in live cells (66).

## Mitochondrial Dysfunction and SCs Organization and Activity in Aging, Alzheimer's Disease (AD) and Parkinson's Disease (PD)

AD and PD are the most frequent neurodegenerative disorders of elderly people with a huge impact on morbidity and mortality. The clinical features of PD are primarily motor deficits, while patients with AD show cognitive impairment. Both conditions are known as proteinopathies, characterized by brain amyloid deposition (abnormal accumulation of misfolded proteins) in specific brain areas and neuronal death. At the histopathological level, the postmortem brain shows similarities and differences in each case. Brains from AD and PD subjects display amyloid deposits (amyloid β and phosphorylated Tau in AD, and α-synuclein in PD), mitochondrial dysfunction and oxidative stress. There is a large amount of experimental evidence indicating that mitochondrial dysfunction is implicated in the pathophysiology of both disorders (67). In this regard, it was proposed that neuronal bioenergetics impairment is a consequence of similar processes in both sporadic disorders. The most relevant alterations include elevated oxidative stress (that can damage mitochondrial respiratory complex expression and/or activity); perturbations in mitochondrial dynamics; alterations in mitochondrial transport within axons; mitophagy; accumulation of somatic mtDNA mutations; impaired quality control mechanisms leading to the accumulation of defective mitochondria; defective calcium (Ca^2+^) homeostasis and signaling. It was postulated that all of these processes may result in neuronal death (68). In post-mortem substantia nigra from PD patients it was found a decrease in CI activity, reduction of ATP levels, increments of ROS and impaired mitochondrial membrane potential leading to Ca^2+^-mediated damage (69), while in the caudate nucleus increased variability in mitochondrial morphology was also observed (70). However, a direct link between CI deficiency caused by mtDNA changes and parkinsonism has not been proven (71). Similarly, post-mortem AD brains show decreased levels of ATP (72) and decreased activity of CIV (73–75). Experimental evidence suggests that mtDNA deletions, which accumulate with age, may be responsible for CIV deficiency observed in AD (76). In addition, reduced levels of CI (77, 78) and CIII (79) were described in agreement with a recent report showing decreased expression of subunits from all respiratory complexes in the entorhinal cortex (80). The process of mitochondrial fusion/fission was also reported to be impaired in AD. Drp1, involved in mitochondrial fission, was found to be reduced in hippocampal post-mortem samples as well as the major proteins responsible for mitochondrial fusion (mitofusins and OPA1) (81, 82). However, until today the mechanisms underlying the correlation between the mitochondria dynamics and SCs assembly have not been defined. Studies carried out in AD and PD models show that alterations in mitochondrial dynamics may lead to an increased production of ROS and SCs disassembly, which in turn may further promote ROS generation and mitochondrial dysfunction. This cause/consequence cycle continuously feeds back, and it is possible that the accumulation of misfolded proteins plays a substantial role in triggering such a process. Experimental evidence supports that both, aging and oxidative stress may influence SCs structure and function. However, a systematic approach to address mitochondrial SCs in the brain of subjects affected with these neurodegenerative disorders or in the brain of animal models of AD and/or PD is scarce. The impact of aging on SCs assembly was addressed by analyzing mitochondria isolated from the cortex of young (5-months-old) and aged (30-months-old) Wistar rats (83). It was observed, using two 2D-BN/SDS-PAGE, decrements of SCs I_1_III_2_ (58% reduction); I_1_III_2_IV_2_ (40.7% reduction) and I_1_III_2_IV_1_ (30.8% reduction), suggesting that aging alters SCs abundance in the cortex, a brain area associated with cognitive functions and particularly affected in AD brains. *In vitro* studies of primary cultures of neurons and astrocytes from rats (Wistar strain) and mice (CBA and C57BL/6 strains) (84) using BN-PAGE followed by in-gel CI activity assay showed that Wistar rats and CBA mice exhibit higher CI activity in astrocytes than in neurons. This can be attributed to the capacity to form SCs containing CIV. In addition, in primary cultures of astrocytes from Wistar rats CI abundance in I-III-containing SCs was higher than in I-III_2_-IV-containing SCs, suggesting that complex IV may determine CI specific activity. Authors suggest that this mechanism could be relevant for neurodegenerative disorders in which lower CI activity has been reported (85). BN-PAGE and the in-gel activity performed in mitochondrial samples isolated from the striatum of 6-hydroxydopamine (6-OHDA) rat model of PD (86), showed that along with the degeneration process, the amount and performance of CI decreased in nearly all forms of SCs. Moreover, CIV activity in SCs (I_1_III_2_IV_3−1_ and I_1_IV_2−1_) progressively decreased during the degeneration process. Furthermore, SCs function has been correlated with mitochondrial membrane plasticity in 6-OHDA-induced dopaminergic neuron degeneration (87). PINK1 (a mitochondrial kinase) and DJ-1 (a redox-sensitive chaperone) loss-of-function mutations are associated with early-onset parkinsonism, and both proteins are involved in maintaining normal mitochondrial dynamics and/or oxidative stress responses (88). Experiments using mitochondria isolated from genetically modified Drosophila showed that impairment on mitochondrial fission mediated by the knock-out of PINK1 causes a decrease in the enzymatic activity and defective assembly of CI and CIV, a significant reduction in mitochondrial respiration, and a reduction in ATP synthesis (20). Furthermore, in mitochondria purified from fibroblasts of patients that carry pathogenic PINK1 mutations, it was observed a decrement in free CI, and in free CI vs. SCs-assembled CI. Free CIII was mildly affected, whereas a decrement of the free CIII vs. SCs-assembled CIII was observed. In the same direction, mitochondria from DJ-1 null dopaminergic neurons showed a decrease in CI formation and activity together with a significant reduction in the abundance of SCs (89). Interestingly, CIV was significantly lower in PD human fibroblasts. These results were also observed in primary cultures of neurons from Pink1-/- mice and in the forebrain of mice lacking DJ1 (Dj1-/-) (90). These observations suggest that OXPHOS complex assembly and function may be modulated by oxidative stress and mitochondrial dynamics, probably involving the fission/fusion machinery (91, 92). Altogether, these findings support that mitochondrial dysfunction in PD may require, among other pathways, the structural remodeling of SCs. Recently, it was shown in postmortem samples of the frontal cortex of AD subjects a significant decrease in the levels of CII, CIII, and CV subunits as compared to controls, while a strong tendency was found for decrements on CIV levels and no differences were detected in CI (93). It is of note that the differences observed by Western blotting were not a consequence of alterations in mitochondrial mass between groups. Moreover, BN-PAGE combined with immunoblotting for subunits of CI and CIII showed that the abundance of respirasomes did not differ between AD and control samples. An overview of the literature on SCs organization and activity in studies using aging, AD and PD models is detailed in Table 1 and a schematic representation of the (dis)assembly of SCs in aging, AD, and PD is shown in Figure 1C. Due to the fact that a few works have been performed to study SCs in AD and PD it became difficult to extract conclusions. More studies are needed, particularly in animal models of age-related neurodegenerative disorders, to clarify to what extent bioenergetics dysfunction at the brain level can be attributed to functional impairment of SCs.

**Table 1:** Overview of the literature on the individual complexes and supercomplex abundance and activity in aging and neurodegeneration.

**Study [References]**	**Model tissue**	**Individual complexes**		**Supercomplex/respirasomes (I-III**_****2****_**-IV**_****n****_**)**	
		**Abundance (WB)**	**Activity (Spectrophotometry)**	**Abundance (BN-PAGE)**	**Activity (*In gel* activity)**
Lopez-Fabuel et al. (84)	Wistar rats, CBA and C57BL/6 mice Primary culture of Astrocytes and Neurons	ND	*Astrocytes* vs. Neurons ↑CI (Wistar and CBA) =CI (C57BL/6) =CIV (Wistar, CBA and C57BL/6)	*Astrocytes* ↑CI free compared to SCs-associated CI (Wistar rats) ↓CI in SCs I-III_2_-IV compared to CI in SC I-III (Wistar rats). *Neurons* =CI free compared to SCs-associated CI (Wistar rats)	*Astrocytes vs. Neurons* = SCs II-III (CBA and C57BL/6) ↓ SC II-III (Wistar)
Frenzel et al. (83)	Aged Wistar rats Brain cortex	↓CIII_2_ ↓CIV	ND	↓ SC III_2_-IV_1_ ↓ SC I- III_2_ ↓ SC I- III_2_- IV_1_ ↓ SC I- III_2_- IV_2_ ↓ SC I- III_2_- IV_3_	ND
Kuter et al. (87)	6-OHDA rat model of PD Stratium 4 days and 4 weeks after lesion	↓CI =CIV	=CI =CIV	↓ CI in SC I-III_2_- IV_n_ = CIV in SC I-III2-IV_n_	= CI in SC I-III2- IV_n_ ↓ CIV in SC I-III_2_-IV_n_ (only 4 weeks after lesion)
Heo et al. (89)	DJ-1 null dopaminergic neurons	↓CI	↓CI	↓ SC I- III_2_ ↓ SC I- III_2_- IV_1_	ND
Kenney et al. (93)	Post mortem human samples Frontal cortex from AD and healthy controls	↓CII ↓CIII ↓CV	ND	= SCs (Mol. Mass > 1.2 mDa)	ND
Lopez-Fabuel et al. (90)	Fibroblasts of patients carrying pathogenic PINK1 mutations Primary culture of Neurons of Pink1-/- mice Neurons and forebrain of DJ1-/- mice	↓CI ↓CI	ND ↓CI (only forebrain mice DJ1-/-)	↓CI free compared to SCs-associated CI = CIII free compared to SCs-associated CIII ↓↓CIV free compared to SCs-associated CIII ↓CI free compared to SCs-associated CI = CIII free compared to SCs-associated CIII ↓↓CIV free compared to SCs-associated CIII	ND ↓CI free compared to SCs-associated CI (only forebrain mice DJ1-/-)

*AD, Alzheimer disease; PD, Parkinson disease; ↓, decrease; ↑, increase; =, no difference; ND, not determined; WB, Western blotting; mDa, mega Daltons*.

## Concluding Remarks

Research on the impact of normal aging and brain amyloid-associated neurological diseases such as AD and PD upon SCs is at a relatively early stage, and therefore many questions remain to be addressed. It is important to take into account that when postmortem frozen tissues are used to study the (dis)assembly of SCs there are at least four critical variables to be considered: (1) the postmortem delay; (2) the freeze-thaw processes that may fracture mitochondria; (3) the stage of disease at which brain samples are analyzed. In advanced stages changes observed in the (dis)assembly of SCs may correspond to the “surviving” neurons and glia while at early stages a more representative picture of neuronal SCs may be obtained; (4) the brain area analyzed. It is very likely that there are differences in SCs assembly in different brain regions because different brain areas are affected in each disorder (hippocampus and cortex are the most affected in AD while striatum is the most affected in PD). Since studies on SCs assembly in different brain areas within each one of these pathologies have not been carried out, this topic remains inconclusive. The convergence of future progress in the study of SCs structure at high resolution and the refinement of animal models of AD and PD as well as the use of iPSC-based and somatic cell-derived neurons will be critical in understanding the biological relevance of the structural remodeling of SCs.

## Author Contributions

GN, PG, and LM: conceptualization. GN, PG, EC, and LM: investigation, writing—original draft, and writing—review and editing. PG and LM: resources and funding acquisition. LM: supervision. All authors contributed to the article and approved the submitted version.

## Conflict of Interest

The authors declare that the research was conducted in the absence of any commercial or financial relationships that could be construed as a potential conflict of interest.
